# Genetic evidence for a recent divergence and subsequent gene flow between Spanish and Eastern imperial eagles

**DOI:** 10.1186/1471-2148-7-170

**Published:** 2007-09-24

**Authors:** Begoña Martínez-Cruz, José Antonio Godoy

**Affiliations:** 1 Estación Biológica de Doñana (CSIC), Avda. María Luisa s/n, 41013 Sevilla, Spain; 2 Unité Eco-Anthropologie et Ethnobiologie, UMR 5145, Musée de l'Homme (Muséum National d'Histoire Naturelle), 17, Place du Trocadéro, 75016 Paris, France

## Abstract

**Background:**

Dating of population divergence is critical in understanding speciation and in evaluating the evolutionary significance of genetic lineages, upon which identification of conservation and management units should be based. In this study we used a multilocus approach and the Isolation-Migration model based on coalescence theory to estimate the time of divergence of the Spanish and Eastern imperial eagle sister species. This model enables estimation of population sizes at split, and inference of gene flow after divergence.

**Results:**

Our results indicate that divergence may have occurred during the Holocene or the late Pleistocene, much more recently than previously suspected. They also suggest a large population reduction at split, with an estimated effective population size several times smaller for the western population than for the eastern population. Asymmetrical gene flow after divergence, from the Eastern imperial eagle to the Spanish imperial eagle, was detected for the nuclear genome but not the mitochondrial genome. Male-mediated gene flow after divergence may explain this result, and the previously reported lower mitochondrial diversity but similar nuclear diversity in Spanish imperial eagles compared to the Eastern species.

**Conclusion:**

Spanish and Eastern imperial eagles split from a common ancestor much more recently than previously thought, and asymmetrical gene flow occurred after divergence. Revision of the phylogenetic proximity of both species is warranted, with implications for conservation.

## Background

The dating of speciation events and investigation of the evolutionary processes involved are major themes in evolutionary biology with important implications for conservation biology. For endangered species, knowledge of these historical processes is critical for correct interpretation of current patterns of genetic variation, and guides assignment of conservation priorities and elaboration of subsequent conservation strategies [1,2].

The classical approach to dating speciation events has been based on the molecular clock, which has proven to be an appropriate tool for addressing a wide range of evolutionary hypotheses [3]. However, several factors have resulted in challenges to the validity of this approach, particularly in application to recently diverged species [4-7]. Firstly, accurate and appropriate calibration points are often lacking, especially for taxa with poor fossil records, including birds [8]. Secondly, the assumptions of rate constancy over time and across lineages are often challenged. Ho and colleagues [9] have reported evidence indicating that the rate of molecular evolution is much faster over short time scales, and thus the timing of many recent events in evolution may have been overestimated. Thirdly, the high variance in coalescent times across loci makes estimation of the divergence time based on a single genetic locus inaccurate, and highlights the need to use multiple unlinked loci. Finally, due to ancestral polymorphism, gene divergence antedates species divergence by a timeframe τ that, in recently separated species, may be very large in relation to the time of split; in birds this difference is typically on the order of several hundred thousand years [10].

New likelihood and Bayesian methods based on coalescence theory have recently been developed for estimating species divergence times, potentially overcoming some of the above handicaps [e.g. [1,11,12]]. Some of these methods apply the Isolation and Migration (IM) model [1,11], which provides estimates of population divergence dates rather than gene coalescence dates, and enables pure isolation with subsequent independent evolution to be distinguished from isolation with subsequent gene flow. The model has been recently extended to make it applicable to data from multiple unlinked loci, even with different modes of inheritance [1].

**Figure 1:**
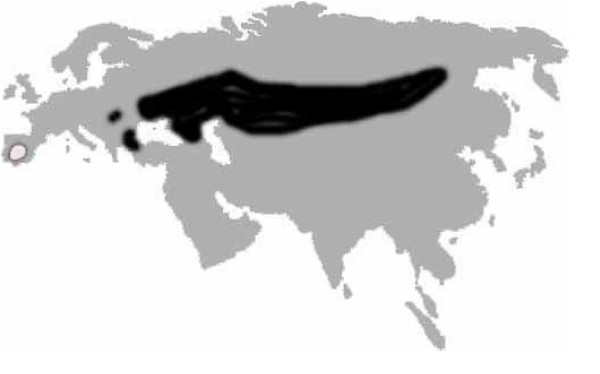
Current worldwide distribution of Spanish and Eastern imperial eagles. The Spanish imperial eagle is confined to the Iberian Peninsula (shaded in light grey), whereas the Eastern imperial eagle has a distribution range through Eurasia (shaded in black).

The status of the Spanish imperial eagle (*Aquila adalberti *Brehm) has fluctuated between threatened and vulnerable in the last 20 years, and it is currently classified as "Vulnerable" in the IUCN red list of threatened species [13]. With less than 200 breeding pairs remaining (Catalogo nacional de especies amenazadas del Ministerio de Medio Ambiente, R.D. 439/1990), the species is confined to the south-western quadrant of the Iberian Peninsula [14]. The population of its sister species, the Eastern imperial eagle (*Aquila heliaca *Savigny), is estimated to be 2,500–10,000 individuals across continental Eurasia. This species is also considered "Vulnerable", although under a less severe criterion [13] (Figure 1). The Spanish imperial eagle has been considered a subspecies (*A. heliaca adalberti*; for example see [15]) of the Eastern imperial eagle because of similarity in diet and the main aspects of their breeding biology [16], and the existence of only minor differences in the adult plumage pattern. However, noticeable differences in juvenile plumages [17] and an allopatric current (though probably parapatric historical) distribution [18] have supported their current designation as separate species [19]. This situation provides an interesting case study of very similar species with large differences in population sizes and distribution ranges. Seibold et al. [20] estimated the date of divergence of these species at around one million years ago (mid-Pleistocene), based on the number of differences in a single mitochondrial gene (*cytochrome b*) and assuming an uncorrected rate (2% My^-1^) of mitochondrial evolution. In this regard it is widely accepted that Pleistocene glaciations were important in shaping the complex pattern of extant species [21-25]. Southern European peninsulas acted as isolated refugia for temperate species during glacial maxima [21,26], allowing the accumulation of variation and divergence through allopatry over an extended time period [21,27]. During interglacial periods species spread from the refugia and recolonized the more northern European areas [21,26]. The ability to fly might have allowed birds to find refugia further south, in Africa. African refugia have been suggested for eastern lineages of *Gypaetus barbatus *[28] and imperial eagles [16].

In the present work we applied a modified IM model [1] to analyse data from an extensive survey of the distribution of *A. adalberti *and *A. heliaca*, in order to investigate the evolutionary processes explaining their divergence. Specifically, we aimed to estimate divergence dates and gene flow following divergence, and assess the implications of our findings for conservation of the endangered Spanish imperial eagle. The results will have implications for the taxonomy of imperial eagles and delimitation of Evolutionary Significant Units (ESUs – defined as genetically differentiated populations that have adaptive differences, and thus merit separate genetic management).

## Results

Estimates of the parameters and values of autocorrelation and ESS (Effective Sampling Size) are listed for the three final runs performed for both sets of markers (Table 1 for mtDNA control region data set and Table 2 for the eight microsatellite dataset). As all runs gave similar results, we report below the estimates from the first run corresponding to the runs which had the highest ESS for the parameter *t *(the parameter with the lowest ESS value in every run).

**Table 1:** Parameter estimates, ESS and autocorrelation values (1 million steps) for each of three runs performed for mitochondrial DNA (mtDNA) dataset

MtDNA dataset		q1	q2	qA	t
Run1	HiPt	5.12	14.86	18.47	0.75
	HPD90Lo	1.53	7.45	7.58	0.27
	HPD90Hi	12.43	26.99	48.66	1.65
	ESS	1601	1266	12901	474
	Autocor	-0.0048	0.014	-0.0029	-0.0066
Run2	HiPt	4.51	14.28	17.58	0.68
	HPD90Lo	1.5	7.52	8.51	0.24
	HPD90Hi	12.37	28.24	39.95	1.66
	ESS	1306	782	16792	349
	Autocor	-0.011	-0.0198	0.001	-0.0493
Run3	HiPt	5.22	14.99	16.52	0.71
	HPD90Lo	1.55	7.34	8.02	0.24
	HPD90Hi	12.55	27.5	43.65	1.76
	ESS	1149	756	14102	348
	Autocor	-0.0133	-0.0039	-0.0002	0.0013

**Table 2:** Parameter estimates, ESS and autocorrelation values (1 million steps) for each of three runs performed for the eight microsatellite dataset

Eight microsatellite dataset		q1	q2	qA	m1	t
Run1	HiPt	1.91	3.58	57.13	0.99	1.35
	HPD90Lo	0.73	1.87	21.88	0.38	0.4
	HPD90Hi	5.43	5.96	170.52	2.47	3.45
	ESS	6808	4136	9630	6256	2315
	Autocor	0.0045	0.0177	0.0073	-0.0004	0.0266
Run2	HiPt	1.91	3.58	56.84	0.99	1.42
	HPD90Lo	0.73	1.87	22.18	0.38	0.4
	HPD90Hi	5.43	5.9	171.11	2.47	3.41
	ESS	6586	4166	10179	5745	1252
	Autocor	0.0019	-0.0035	0.0003	0.0108	-0.0087
Run3	HiPt	1.91	3.64	56.55	1	1.37
	HPD90Lo	0.73	1.87	22.47	0.36	0.41
	HPD90Hi	5.43	5.9	169.94	2.53	3.35
	ESS	6194	4652	11189	5141	2495
	Autocor	-0.0046	-0.0104	-0.0046	0.002	-0.0244

Microsatellite maximum likelihood estimates of population sizes were θ_1 _= 1.9 (0.73–5.43, 90% Highest Posterior Density, HPD), θ_2 _= 3.6 (1.87–5.96, 90% HPD) and θ_A _= 57.1 (21.88–170.52, 90% HPD), and for the mitochondrial marker were θ_1 _= 5.12 (1.53–12.43, 90% HPD), θ_2 _= 14.86 (7.45–26.99, 90% HPD) and θ_A _= 18.47 (7.58–48.66, 90% HPD). Our estimates for θ_A _suggest a bottleneck for both populations at the time of split from the common ancestor, and that this was more intense in the case of *A. adalberti*.

**Figure 2:**
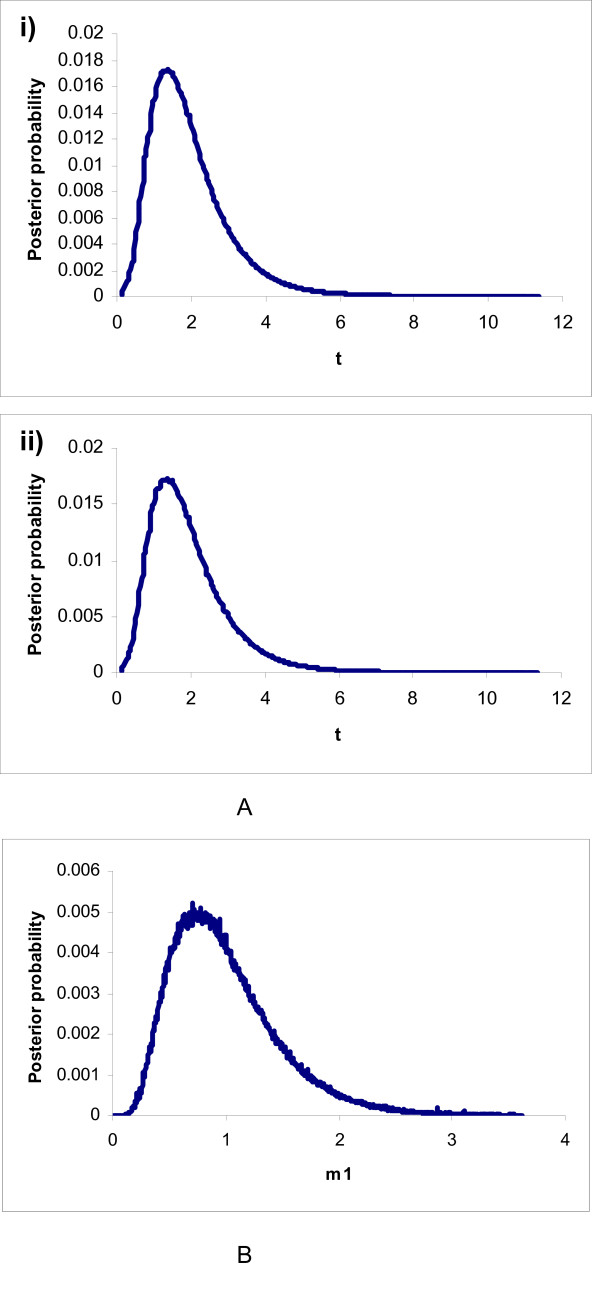
Posterior probability distributions. A) Distributions for *t *for i) the set of eight microsatellite markers, under an SMM model and ii) the mitochondrial dataset, under the HKY model. B) Distributions for *m*_*1 *_for the set of eight microsatellite markers, under an SMM model.

Posterior probabilities for the divergence times are plotted in Figure 2A for both marker types. In the case of the microsatellite data set, the posterior probability distribution peaked at time *t *= 1.35 (0.40–3.45, 90% HPD). The estimate of *t *for the mitochondrial data reached a maximum probability at *t *= 0.75 (0.27–1.65, 90% HPD). Because *t *is scaled by the mutation rate μ, and θ = 4N_e_μ, an estimate of time in units of 2N_e _generations can be obtained by dividing the ML estimate of *t *by one half the ML estimate of θ. For the nuclear genome we obtained an estimate of 1.4 × 2N_e _generations (0.4–3.6, 90% HPD), and for the mitochondrial D-loop the estimate was 0.29 × 2N_e _generations (0.11–0.64, 90% HPD). The N_e _harmonic mean for *A. adalberti *during the last century was estimated for 117 individuals (B. Martínez-Cruz and J.A. Godoy, unpublished data) using a likelihood-based temporal approach [29]. Assuming a mean generation time of 16.4 years [30], this results in divergence time estimates of 5.37 × 10^3 ^years ago (1.54 × 10^3^–1.38 × 10^4 ^years ago, 90% HPD) for the nuclear genome, and 1.11 × 10^3 ^years ago (422–2.46 × 10^3 ^years ago, 90% HPD) for the mitochondrial genome. As we estimated N_e _for a period when the *A. adalberti *population had undergone a decline, the value obtained might be an underestimate of the historical population. However, ecological constraints suggest that the population has never been large [16], and in a previous study we suggested that the demographic bottleneck was not strong enough to affect genetic diversity [31]. Even with a reduction up to 20% of the historical population size (historical N_e _around six hundred), the estimated time of divergence would be 2.69 × 10^4 ^years ago (7.7 × 10^3^–6.9 × 10^4 ^years ago, 90% HPD) for the microsatellite marker, and 5.6 × 10^3 ^years ago (2.30 × 10^3^–1.29 × 10^4 ^years ago, 90% HPD) for the mitochondrial marker. Calculation of divergence time from *t *and μ produced similar results. For the microsatellite marker, assuming a mean value of 5 × 10^-4 ^for microsatellite mutation rates [32], the estimate of time of divergence is 2.9 × 10^3 ^years ago. For the mitochondrial marker the estimated time of divergence is 5.98 × 10^2 ^years ago, assuming that the mitochondrial control region evolves 10.4 times faster than the entire mitochondrial genome [33], and thus a mean rate of evolution of 20.8 % My^-1 ^for this fragment.

Gene flow is suggested by the microsatellite data but not the mitochondrial data, indicating that recurrent gene flow occurred between the two eagle populations, from *A. heliaca *to *A. adalberti*. We ran the model setting *m*_2 _to zero (estimating five parameters: *θ*_*1*_, *θ*_*2*_, *θ*_*A*_, *m*_*1 *_and *t*). The log likelihood ratio log [max{L (m_1 _= 0 | X)}/max{L (m_1 _| X)}] was -3.12, suggesting the adequacy of a model that includes migration. The proportion of *A. adalberti *replaced by *A. heliaca *migrants, scaled by mutation rate, was estimated in *m*_1 _= 0.99 (0.38–2.47, 90% HPD) (Figure 2B). If the relationship 2N_1_m_1 _= 4N_1_μ × 1/2 × m_1_/μ [34] and estimates of θ and μ are used, an estimated migration of 0.94 gene copies per generation is obtained. Therefore, our analysis suggests that approximately one Eastern male imperial eagle might have effectively reached the Spanish imperial eagle population per generation since they started to diverge.

## Discussion

Analysis of two independent data sets indicated that the Eastern and Spanish imperial eagles split from a common ancestor much more recently than has been suggested [16,20], and that the split occurred during the Holocene or the very late Pleistocene. The absence of imperial eagle remains in the Iberian fossil record for the entire Pleistocene is consistent with the hypothesis that colonization of the Iberian peninsula was very recent, on the order of several thousand years (A. Sánchez Marco, personal communication), but contradicts the previous phylogenetic estimate of one million years ago for the divergence of these species [16,20]. Estimates of the population parameter θ for the ancestral and descendant populations suggest that the split would have significantly reduced the size of a large ancestral population, affecting the western descendant population to a greater extent. A recent divergence and a large effective size of the ancestral population could explain the large differences in the time of split estimated by phylogenetic and coalescent-based methods. Moreover, the recent work of Ho et al. [9], which showed that rate of change for molecular divergence for recent evolutionary events (< 2 million years) is much higher than the traditionally accepted 2% per million years [9], would suggest that the phylogenetic estimate of one million years ago for the divergence of the two eagles is a substantial overestimation. These results also suggest the need for revision of the dating of speciation events in birds, which are often dated to the early or middle Pleistocene by phylogenetic methods, and brings into question the importance of glaciation during the Pleistocene in the diversification of bird species [2,35,36]. In the case of imperial eagles, such a recent divergence also raises questions concerning their current taxonomic status as separate species, originally proposed primarily on the basis of plumage patterns [17]. In this regard the occurrence of *A. heliaca *(Eastern) plumages in both adult and juvenile *A. adalberti *individuals, observed in the field at low frequencies (Spanish Ministry of Environment, unpublished data), might indicate retained ancestral polymorphism consistent with a recent separation of imperial eagles.

If imperial eagles remained isolated during repeated glacial maxima in two different glacial refugia [16], our data would suggest that divergence was subsequently aborted by secondary contacts during interglacial periods, until they were finally differentiated during the last glaciation. Alternatively, divergence could have occurred through a recent colonization of Iberia from Africa, as suggested for other steppe birds [37], followed by disruption of its distribution range in northern Africa. This colonization might have occurred, as there was suitable steppe habitat on the Iberian Peninsula 10,000 years ago during the Younger Dryas period, as revealed by palynological data [38]. Movement into the Iberian Peninsula could have occurred from the north of Africa, where the Spanish imperial eagle existed until last century [39]. Up to 80% of bird species in northern Africa are of Palaearctic origin [40], and the semiarid to subhumid conditions during the Neolithic were probably suitable for imperial eagles in this area, while the presence of a more or less dense tree cover throughout Europe may not have provided suitable habitat. More recently, the aridity in North Africa since 3,000 B.P. could have reduced animal populations to refuges [40], and disrupted the distribution range of the imperial eagle. A detailed study of the phylogeography of the species, specifically including museum samples from northern Africa, is necessary to test this hypothesis.

Our results also indicate male-mediated gene flow from Eastern imperial eagles to Spanish imperial eagles after divergence. This may have occurred by eastern eagles deviating in their wintering routes to and from Africa through continental Italy and Sicily [18], and finally wintering in Spain. This phenomenon has been described for other raptor species including *A. clanga *(spotted eagle) and *A. pomarina *(lesser spotted eagle) (Monitoring Group of the Doñana Biological Station, unpublished data). The absence of female-mediated gene flow could also be a consequence of the lower fitness of heterogametic female hybrids (following Haldane's rule [41]), as has been suggested for *A. clanga *and *A. pomarina *[42] and documented for other species [43,44]. Male mediated asymmetrical gene flow could explain the low mitochondrial but high nuclear diversity in Spanish imperial eagles (both currently and in the population predating the decline which occurred during the 20th century [31,45]), as well as the reciprocal monophyly observed for the mitochondrial genome. Therefore, reduced mitochondrial diversity, far from being a consequence of recent demographic alterations, seems to be a signature for a founder effect, a history of small N_e _or restricted female-mediated gene flow.

The recent split and occurrence of subsequent gene flow have implications for the taxonomic and conservation status of both imperial eagles. Although the asymmetric gene flow pattern might still indicate some level of reproductive incompatibility, full reproductive isolation is contradicted by our observations of gene flow. Based on the scarcity of diagnostic characters (such as the *A. heliaca *plumages in both adults and juveniles occurring in the *A. adalberti *population; Spanish Ministry of Environment, unpublished data) and the low level of morphological, ecological or behavioural divergence, these taxa might be conservatively classified as allospecies based upon the criteria of Helbig et al. [46]. Finally, our results preclude rejection of historical genetic exchangeability [47], and highlight the need to determine whether both allospecies represent different ESUs by evaluating current ecological exchangeability, ideally through an experimental approach [48]. If exchangeability cannot be ruled out, and if the Spanish imperial eagle viability were to be compromised anew, the introduction of Eastern individuals should be carefully considered [48].

## Conclusion

Our results have shown that the split that gave rise to Spanish and Eastern imperial eagles was very recent, and that asymmetrical gene flow occurred after divergence. This presents a completely unexpected scenario for the evolutionary origins of imperial eagles, with implications for management strategies for the endangered *A. adalberti*. Our work also shows the potential of coalescent-based models in inferring evolutionary processes, and illustrates the value of an historical perspective in developing a full understanding of contemporary genetic patterns, and in design of adequate conservation strategies.

## Methods

### Samples, sequencing, genotyping and data analysis

Thirty-four museum specimens of *A. adalberti*, representing the population one hundred years ago [45], 33 contemporary *A. heliaca *individuals [31], and 50 museum specimens of *A. heliaca *were analysed for a 345 bp mitochondrial control region fragment (EMBL accession numbers AJ567366, AJ567367, AJ937835 and AJ574878–AJ574885). A random subset of 15 individuals of each species was analysed for eight microsatellite loci (GenBank accession numbers AF469499, AF469502, AF469504, AF469505, AF469507, AF469509, AF469510 and AF469512) [49]. The *A. adalberti *historic sample was included to avoid the possible influence of the recently arisen genetic structure in the analysis [45]. Large allelic frequency fluctuations across generations are unlikely for *A. heliaca *in the period analysed, as the species has always been globally abundant and widely distributed. A complete description of the samples is available upon request.

The basic IM model [11,50-52], as modified by Hey and Nielsen [1] to make it applicable to data from multiple unlinked loci, was used in this study. Six different parameters can be estimated in this coalescent model, in which an ancestral population (size N_A_) splits at time t into two descendant populations (sizes N_1 _and N_2_) which may subsequently establish gene flow between them at rates m_1 _and m_2 _(the proportion of populations 1 and 2 that are replaced by migrants from the other population, respectively). The model assumes selective neutrality and no further population subdivision within each population. It also assumes that population sizes do not change over time. Nielsen and Wakeley [11] implemented a likelihood/Bayesian approach that uses a Metropolis-Hastings, Markov Chain Monte Carlo algorithm. Estimates of the parameters were based on the posterior distribution of those parameters, p (T, M, θ | X), where T is the time of split, M is the migration rate that may be asymmetrical (that is m_1 _≠ m_2_), θ is the population parameter 2Neμ, and X is the data. It was necessary to apply some assumptions to these parameters, described by the prior distributions, to estimate the posterior distribution. Firstly, we assumed that all parameter values were equally likely (uniform distribution), so that posterior probabilities are proportional to the likelihood function. Secondly, to ensure that the posterior distribution was a real probability distribution, we constrained the parameter space, i.e. we set maximum values for the priors of the parameters. By doing so, the results can be interpreted in a Bayesian framework as well as in a likelihood framework. The model was modified [52] using the HKY model of molecular evolution to include the possibility of multiple mutations in the same site [53]. More recently Hey and Nielsen [1] included a stepwise mutation model, and a procedure for considering loci that combines both an infinite sites component and a stepwise component.

A critical issue with the use of MCMC approach is whether the parameter space has been fully explored and the stationary distribution has been adequately sampled. As recommended by Hey and Nielsen [1] we first performed multiple runs for each set, with an increasing number of steps and using wide priors to ensure that the complete posterior distribution could be obtained. Low autocorrelation parameters (i.e. < 0.03), parameter update rates greater than 2%, and similar distributions after several independent runs were used as criteria for adequate mixing. We found multiple heated chains, implemented in the program, did not perform as well as a single chain based on these criteria. All six parameters were estimated for both datasets. For the microsatellite analysis, the full model of six parameters resulted in zero migration from the Spanish into the Eastern imperial eagle population; for the mitochondrial analysis, the full model of six parameters resulted in zero migration for both, m1 and m2. Hypothesis testing on the significance of migration values was performed for both marker types by comparing the likelihood value at m = 0 to the likelihood value obtained at the maximum likelihood estimate, and contrasting the log likelihood ratio against the distribution of the simulations previously presented [Figure 5 in ref [11]]. In cases where migration was not different from zero, the full model was run again with those parameters set to zero (m_2 _for the microsatellite dataset, and both m_1 _and m_2 _for the mitochondrial dataset). We finally performed three independent runs of 5 × 10^8 ^steps with a burn-in of 2 × 10^6 ^steps for the set of eight microsatellite markers, and three independent runs of 2 × 10^7 ^steps with a burn-in of 1 × 10^6 ^steps for the control region sequence dataset, using narrow priors after results of the preliminary analyses.

Analyses were performed in a Pentium 4, 3.2 GHz, 1 GB processor integrated in a supercomputer with 80 DELL 750 nodes interconnected by Gigabit Ethernet to a data server 1750 with two processors, Xeon 3 GHz and 438 GB SCI disk, in RAID 5 configuration at the Centro de Supercomputacion de Galicia (CESGA), Spain. Each of the three final runs for each marker took around 138 hours for the microsatellite dataset and around 27 for the mtDNA dataset in a single processor.

## Competing interests

The author(s) declares that there are no competing interests.

## Authors' contributions

BMC carried out the genotyping of individuals, analysed the microsatellite raw data and performed the analysis with the IM software. BMC and JAG conceived and elaborated the design of the study. JAG coordinated the study, supervised the drafting of the manuscript and read and approved the final manuscript. All authors have read and approved the final manuscript.
